# Antiproliferative and Apoptotic Potential of Cyanidin-Based Anthocyanins on Melanoma Cells

**DOI:** 10.3390/ijms18050949

**Published:** 2017-04-30

**Authors:** Dumitriţa Rugină, Daniela Hanganu, Zoriţa Diaconeasa, Flaviu Tăbăran, Cristina Coman, Loredana Leopold, Andrea Bunea, Adela Pintea

**Affiliations:** 1 Faculty of Veterinary Medicine, University of Agricultural Sciences and Veterinary Medicine Cluj-Napoca, Mănăştur Street 3-5, 400372 Cluj-Napoca, Romania; dumitrita.rugina@usamvcluj.ro (D.R.); flaviu_tabaran@yahoo.com (F.T.); 2 Faculty of Pharmacy, “Iuliu Haţieganu” University of Medicine and Pharmacy, Victor Babeș Street 8, 400012 Cluj-Napoca, Romania; danahcluj@gmail.com; 3 Faculty of Food Science and Technology, University of Agricultural Sciences and Veterinary Medicine, Mănăştur Street 3-5, 400372 Cluj-Napoca, Romania; zorita.sconta@usamvcluj.ro (Z.D.); cristina.coman@usamvcluj.ro (C.C.); loredana.leopold@usamvcluj.ro (L.L.); 4 Faculty of Animal Science and Biotechnology, University of Agricultural Sciences and Veterinary Medicine, Mănăştur Street 3-5, 400372 Cluj-Napoca, Romania; andrea_bunea@yahoo.com

**Keywords:** anthocyanins, apoptosis, melanoma

## Abstract

Elderberries are known for their high anthocyanins content, which have been shown to possess anti-proliferative and anti-cancer effects. Anthocyanins enriched extract (AEE) was obtained from elderberries and was characterized by LC/DAD/ESI-MS analysis. Five cyanidin-based anthocyanins were identified, among which Cy-3-*O*-samb was the major compound (51%). The total anthocyanins content of AEE was 495 mg Cy-3-*O*-samb/100 g FW. AEE inhibited proliferation of metastatic B16-F10 murine melanoma cells, in a concentration-dependent manner, with an IC50 of 264.3 μg/mL. LDH (lactate dehydrogenase), as a marker of membrane integrity, increased 74% in B16-F10 cells treated with 250 μg/mL AEE, compared to control. It was observed that apoptosis is the mechanism of melanoma cell death after AEE treatment, confirmed morphologically by acridine orange/ethidium bromide double staining and TUNEL analysis. These results indicate that elderberry-derived anthocyanins might be utilized in future applications as topical adjuvant in skin cancer therapy.

## 1. Introduction

Plants contain compounds with photoprotective or anti-cancer potential that could be exploited as new drugs. Recently, compounds extracted from natural sources are gaining in popularity, because they exhibit potential as topical agents in the treatment or prevention of skin cancer. Anthocyanins have been shown to confer skin photo-chemopreventive or anti-cancer protection [1,2,3,4,5,6,7]. In vivo studies on SKH-1 hairless mice, a model of UVB-induced skin carcinogenesis, showed that Cy-3-*O*-gluc reduced glutathione level and decreased the production of pro-inflammatory cytokines (IL-6 and TNF-α) and phosphorylated MAP kinases, ERK1/2, p38, JNK1/2 and MKK4 [7]. Protective effects of delphinidin against UVB-induced apoptosis were previously investigated on immortalized HaCaT keratinocytes cell line and SKH-1 hairless mouse model, respectively [1]. The protective effects of cyanidin and delphinidin toward epidermal skin JB6 P+ cells exposed to UVB-induced irradiation, via suppressing the transactivation of transcription factors (NF-κB and AP-1) and via regulation of MAP kinases, were also previously reported [5,8]. In primary keratinocytes exposed to UV-mediated oxidative damage, blackberry extracts upregulated the expression of antioxidant enzymes as catalase, manganese superoxide dismutase, gluthatione peroxidase, and gluthatione *S*-transferase A1 [6].

While a lot of encouraging data has been generated regarding anthocyanins, research has yet to focus on the pharmacological properties of these compounds, such as bioavailability, bioabsorption, uptake, mechanism of action, and tissue localization. With respect to elderberry anthocyanins bioavailability, literature data report the bioabsorption of anthocyanins approximately 2 h after consumption and their excretion in urine within 5 h [9,10]. Nevertheless, scientific data sustains that only 1% of consumed anthocyanins arrive in plasma, subsequently in urine [9,11,12]. Therefore, direct administration of anthocyanins on tumor skin cells can potentially overcome disadvantages such as their low bioavailability, uncontrolled release, long blood circulation time, non-selective tissue distribution, and high dose needed in oral administration.

The present study, designed on a melanoma mouse skin cellular model, provides novel preliminary data on inhibition of cell proliferation and induction of apoptosis by cyanidin-based anthocyanins isolated from *Sambucus nigra* fruits. Our findings suggest that these compounds might be recommended as agents topically applied to the skin related cancers.

## 2. Results and Discussion

### 2.1. LC/DAD/ESI-MS Analysis of Anthocyanins Enriched Extract (AEE)

**Figure 1:**
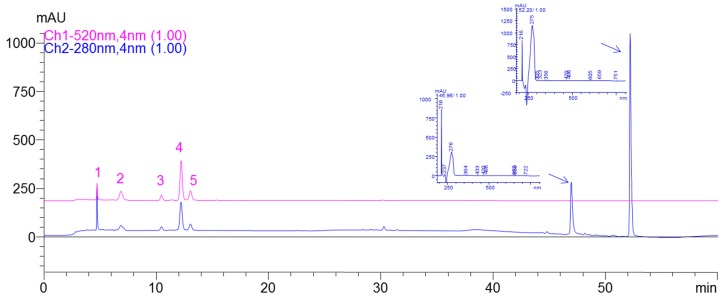
Typical analytical LC-DAD chromatogram of anthocyanins enriched extract (AEE) extract recorded at 520 nm, respectively at 280 nm. Identified peaks at 520 nm correspond to Cy-3-*O*-samb-5-gluc (peak 1), Cy-3,5-digluc (peak 2), Cy-hexoside-pentoside (peak 3), Cy-3-*O*-samb (peak 4), and Cy-3-*O*-gluc (peak 5). Unidentified peaks from chromatogram recorded at 280 nm are compounds found in the AEE, after SPE (solid phase extraction) clean-up procedure, more likely phenolic acids.

UV spectra from LC/DAD/ESI-MS analysis of anthocyanins enriched extract (AEE) showed that peaks with typical anthocyanin absorption spectra were eluted at 4.6, 6.8, 10.4, 12.2, and 13 min and correspond to: Cy-3-*O*-samb-5-gluc (peak 1); Cy-3,5-digluc (peak 2), Cy-hexoside-pentoside (peak 3), Cy-3-*O*-samb (peak 4), and Cy-3-*O*-gluc (peak 5) (Figure 1).

The data of the mass spectra indicated that the molecular structure of anthocyanins matched with cyanidin derivatives, based on the presence of the typical ion at *m*/*z* 287, characteristic of the cyanidin aglycon. Peak (1) was confirmed to be Cy-3-*O*-samb-5-gluc, with the molecular cation [M^+^] at *m*/*z* 743, and the fragment ions *m*/*z* 581 (M^+^-162, corresponding to the loss of one glucosyl unit), and respectively *m*/*z* 449 (consecutive loss of xylosyl unit) and 287 (the loss of the second glucosyl unit). The second peak (2) was identified as Cy-3,5-digluc, having the molecular cation [M^+^] at *m*/*z* 611, and fragment ions at *m*/*z* 449 ([M^+^]-162) and 287. Peak 3 presented the molecular cation [M^+^] at *m*/*z* 581 and fragment ions at *m*/*z* 449 and respectively 287, correspond to a Cy-hexoside-pentoside. Cy-3-*O*-samb was identified as the major compound (peak 4), presenting molecular cation [M^+^] *m*/*z* 581, and fragment ions at *m*/*z* 449 and 287. It represents 51% of total anthocyanins in AEE followed by Cy-3,5-digluc (17%). Cy-3-*O*-gluc (peak 5), characterized by the molecular cation [M^+^] at *m*/*z* 449 and the fragment ion *m*/*z* 287, represents the third important compound (14%) in AEE. It can be seen in the chromatogram recorded at 280 nm that AEE contains impurities, which could be phenolic acids according to their UV-VIS spectrum.

Total anthocyanins content was 495.16 mg Cy-3-*O*-samb/100 g FW. The average content in AEE of Cy-3-*O*-samb-5-gluc (48.49 mg/100 g), Cy-3,5-digluc (24.9 mg/100 g), Cy-hexoside-pentoside (35.34 mg/100 g), Cy-3-*O*-samb (255.56 mg/100 g), and Cy-3-*O*-gluc (71.18 mg/100 g) was expressed in Table 1 as mg Cy-3-*O*-samb/ 100 g of fresh fruit (FW).

### 2.2. Melanoma Cell Proliferation

**Figure 2:**
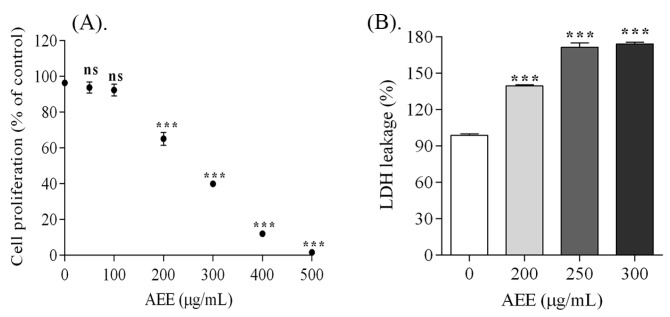
Antiproliferative and cytotoxic effects of AEE on B16-F10 murine melanoma cells, after 24 h. B16-F10 cells proliferation effect AEE-induced by different concentrations (0–500 μg/mL) (**A**). Stimulation of lactate dehydrogenase (LDH) release by AEE-induced membrane injury in B16-F10 cells (**B**). The data were expressed as mean ± standard error of mean (SEM) from three replicates for each experiment, each experiment having five replicates for each sample. Statistically extremely significant differences: *** *p* < 0.001 compared with control; Non statistically significant—ns.

The effect of AEE on murine melanoma B16-F10 cells proliferation was determined by performing MTT assay. As shown in Figure 2, the treatment with AEE (50, 100, 200, 300, 400, 500 μg/mL; 24 h) applied on B10-F10 cells reduced cell proliferation in a dose-dependent manner. AEE treatment resulted in approximately 35%, 60% and 88% decrease in cell proliferation at 200, 300 and 400 μg/mL respectively. Based on the IC50 value, three doses (200, 250, and 300 μg/mL) were chosen for further experiments to evaluate the cytotoxic and apoptotic effects of AEE.

### 2.3. Assessment of Melanoma Cells Membrane Integrity

**Figure 3:**
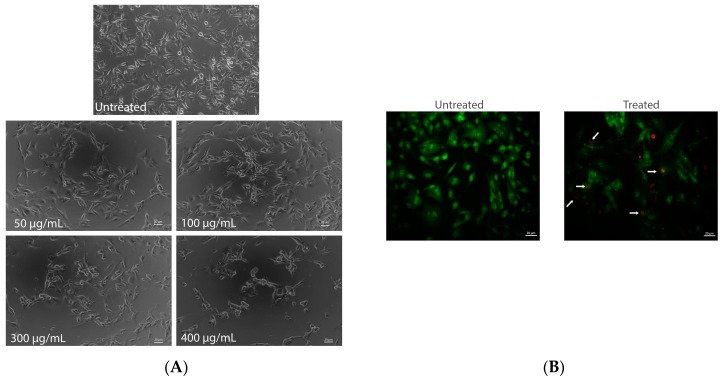

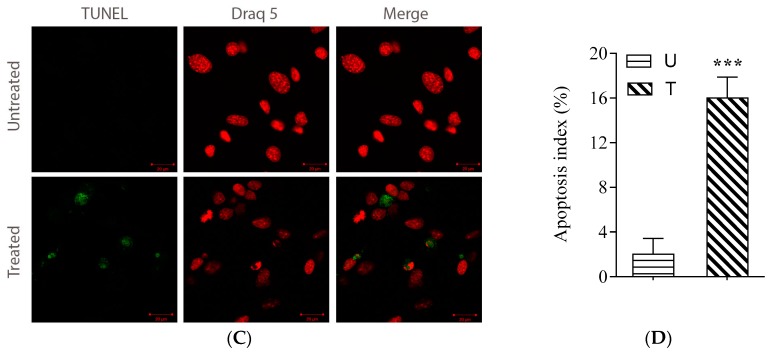
Induction of apoptosis by AEE extract. Contrast-phase microscopy on B16-F10 murine melanoma cells was untreated and treated with AEE, in coverslides, for 24 h (**A**). Fluorescence microscopy by dual staining AO/EB (acridine orange/ ethidium bromide) of B16-F10 cells. Untreated cells and cells with intact membrane appeared colored green, being permeable for EB. B16-F10 cells treated AEE (250 μg/mL) labeled with both dyes simultaneously, apoptotic cells appeared orange (indicated with white arrows) (**B**). TUNEL assay for apoptosis detection. B16-F10 cells were treated with AEE (250 μg/mL) for 24 h and analyzed for apoptosis using an in situ cell death kit ApopTag^®^ Red in situ Apoptosis Detection kit (Chemicon, Millipore, Billerica, MA, USA). TUNEL-positive cells are shown as green fluorescence; normal nuclei are stained red with Draq5 (**C**). Quantification of TUNEL assay. Data are presented as percentage of TUNEL-positive cells per 1000 cells; U-untreated cells; T-AEE treated cells (**D**).

In order to detect the cytotoxic effect of AEE, total lactate dehydrogenase (LDH) release was assessed. A concentration-dependent increase of total secreted LDH proves that the membranes of B16-F10 cells were strongly affected by 200, 250 and 300 μg/mL AEE doses. AEE (250 μg/mL) induced an increase of LDH with 74% by compared to control after 24 h (Figure 3).

### 2.4. Evaluation of Apoptotic Cell Death

B16-F10 cells were treated with AEE (250 μg/mL) and found to induce chromatin condensation/fragmentation. Apoptosis was confirmed by contrast-phase microscopy, fluorescence microscopy (dual staining AO/EB) and confocal microscopy (TUNEL assay). According to the contrast-phase microscopy images, apoptosis characteristics—such as detachment, rounding up, shrinkage, and blebbing of membrane and apoptotic bodies—were seen in B16-F10 cells exposed to AEE concentrations higher than 200 μg/mL, for 24 h (Figure 3A).

In order to establish the integrity of cellular membranes, a dual staining with EB and AO dyes was used. The cells with intact membrane appear colored green, due to AO-selective permeability. AEE treatment (250 μg/mL) affected the integrity of B16-F10 cell’s membranes, therefore membranes also became permeable for EB dye. This resulted in condensed and fragmented chromatin labeled with dual dyes simultaneously—EB and AO—and cells appeared orange in color (Figure 3B). More than 1000 treated and non-treated cells were analyzed and counted. About 18% of melanoma cells treated with AEE were colored orange and considered to be in apoptosis comparing to untreated cells, colored green. Some cells (less than 3%) were colored red, suggesting a late stage of apoptosis. Furthermore, we analyzed whether AEE treatment affected DNA by using simultaneously an end labeling assay (TUNEL) and Draq5 nuclei staining. It is known that DNA double stranded cleavage occurs during apoptosis, resulting in DNA fragments like single strand breaks (nicks). The DNA single strand breaks, were labeled at the free 3′-OH termini with modified nucleotides (green) and observed by confocal microscopy (Figure 3C). In contrast, normal or proliferative nuclei, which have a relatively insignificant number of DNA 3′-OH ends, usually do not stain. AEE treatment (250 μg/mL) lead to a 16% increase in TUNEL positive cells, compared to their corresponding control cells, where the percent of TUNEL positive cells was about 2% (Figure 3D).

## 3. Discussion

Anthocyanins are the pigments responsible for the red, purple, and blue colors of most fruits and vegetables, which have been associated with protective effects against many chronic diseases including cancer. Over the past decade, many researchers investigated the potential health benefits of anthocyanins from fruits and vegetables, without eliminating other bioactive compounds from crude anthocyanin extracts. In this study, we tried to eliminate other bioactive compounds from elderberry crude anthocyanin extract by exploring the use of a low-cost anthocyanin fractionation technique as a way to obtain an enriched anthocyanin extract. Even if the fractionation method used in this study was suitable to obtain an enriched cyanidin-based anthocyanins fraction, according to the LC/DAD chromatogram at 280 nm, AEE proved to contain impurities. Nevertheless, the recorded LC/DAD chromatogram at 520 nm shows that AEE consists of five anthocyanins: Cy-3-*O*-samb-5-gluc, Cy-3,5-digluc, Cy-hexoside-pentoside, Cy-3-*O*-samb, and Cy-3-*O*-gluc. A similar profile of anthocyanins have been previously reported in *Sambucus nigra* fruits, containing Cy-3-*O*-samb, Cy-3-*O*-gluc, and Cy-3,5-digluc as major anthocyanins [13,14]. Additionally to the above mentioned compounds, Wu et al. (2004) identified small amounts of Cy-3-*O*-rut, Pelarg-3-*O*-gluc, and Pelarg-3-*O*-samb in American elderberries [15]. In contrast to European elderberries, the same study reported that the major compound in American elderberries was Cy-3-*O*-gluc (739 mg/100 g FW), followed by Cy-3-*O*-samb (545 mg/100 g FW). They reported a total amount of anthocyanins (1374 mg/100 g FW) higher than those usually found in Europeans hybrids [13,16,17]. Our quantitative data on total anthocyanins isolated from AEE are consistent with those previously found in Europeans hybrids (495.16 mg/100 g FW) [16,17,18]. Differences in the total amount or in the ratio between individual anthocyanins reported in these studies could be due to the influence of genetic and environmental factors or to differences in extraction/fractionation procedures.

To our knowledge, this is the first study to evaluate the antiproliferative action and the apoptotic potential of isolated elderberry anthocyanins fraction on melanoma cells. Our data showed that AEE significantly inhibits the proliferation of murine melanoma cells in a concentration dependent manner. The morphological features of AEE-treated melanoma cells also confirmed these results.

The antiproliferative potential of AEE carried out on B16-F10 cells showed positive results. Therefore, AEE extract could be pharmaceutically active at doses ≤ 264.3 μg/mL. Moreover, B16-F10 cells membranes were strongly affected by 200, 250, and 300 μg/mL AEE doses after 24 h, as proved by the increase of LDH release. Cyanidin-based anthocyanins from AEE seemed to have stronger effects than anthocyanins isolated from blueberries. Higher doses of anthocyanins of 550, 600, and 650 μg/mL isolated from blueberries applied on B16-F10 cells were necessary to decrease cell proliferation and to increase the secreted LDH with 9%, 10% and 12% [19]. A recent paper reported that anthocyanins extracted from bog bilberry increased LDH release in Caco-2 cell line by 21% after 48 h and in Hep-G2 cell line by 66% at 48 h [20].

Induction of apoptosis is the key to prove that AEE could be a potential anticancer agent. After 24 h of treatment with AEE (250 μg/mL), the characteristics of apoptotic cells as DNA fragmentations was observed, increasing TUNEL positive cells by 16%. In our previous study, blueberry anthocyanins (600 μg/mL) stimulated apoptosis, increasing the TUNEL positive melanoma B16-F10 cells by 14% compared to control [19]. Therefore, all these data suggest that anthocyanins could induce apoptosis in treated B16-F10 murine melanoma cells. Additionally, blueberry anthocyanins were able to increase DNA fragmentation by 2–7 times in HT-29 and Caco-2 cells [21]. Anthocyanins form other sources, as red potato, induced apoptosis in LNCaP and PC-3 cells, as demonstrated by TUNEL assay [22].

The anti-cancer activity of anthocyanins could be linked to some of their structural features. Therefore, it appears that the presence of acylated acids or increased number of sugar moieties reduces the anti-cancer activity of anthocyanins. The structure-function relationships of anthocyanins were recently reviewed [23]. Non-acylated anthocyanins isolated from different blueberry and *Cornus kousa* fruits showed great inhibition against B16-F10 (melanoma), MCF-7 (beast), SF-268 (CNS), NCI-H460 (lung), HCT-116 (colon), and AGS (gastric) human tumor cells tumor cell lines [19,24]. Non-acylated anthocyanins from sweet potato—containing Cy-3-*O*-sophoroside-5-gluc—proved to have an anti-cancer activity in human promyelocytic leukemia HL-60 cells [25]. Additionally, monoglycosylated cyanidin derivatives of chokeberries inhibited the growth of human colorectal adenocarcinoma HT-29 and human cervix adenocarcinoma HeLa cells [26,27]. Recently, nonacylated monoglycosylated anthocyanins were found to have a greater anti-inhibitory effect on HT-29 cells than triglycosilated anthocyanins and/or anthocyanins acylated with cinnamic acid [28]. In our current study, cyanidin-based anthocyanins isolated from elderberries are nonacylated monoglycosylated structures and proven to exert anti-proliferative and anti-apoptotic activities toward murine melanoma cells. Nevertheless, the relationship between anthocyanin structure and their anti-cancer potential remains a topic of our further research.

## 4. Materials and Methods

### 4.1. Reagents

The standard compounds—including Cy-3-*O*-samb-5-gluc chloride, Cy-3-*O*-samb chloride, Cy-3-*O*-gluc, and Cy—were purchased from Extrasynthese (Lyon, France). Water used for current experiments was treated in a Milli-Q water purification system. In Vitro Toxicology Assay Kit (LDH based TOX7), glutamine, penicillin, and streptomicin were purchased from Sigma Chemical Co. (St. Louis, MO, USA). Fetal bovine serum (FBS), Dulbecco’s Modified Eagle Medium (DMEM), and 3-(4,5-Dimethylthiazol-2-yl)-2,5-diphenyltetrazolium bromide (MTT) were purchased from Lonza Group Ltd. (Basel, Switzerland). ApopTag Red In Situ Apoptosis Detection Kit was aquisitioned from Chemicon (Millipore, Bedford, MA, USA) and Draq5 was purchased from Cell Signaling Technology, Inc. (Beverly, MA, USA). Methanol (MeOH), ethyl acetate (EtOAc), formic acid, acetonitrile, hydrochloric acid (HCl), dimethylsulfoxide (DMSO), ethidium bromide/ acridine orange (EB/AO), and paraformaldehyde were bought from Merck (Darmstadt, Germany).

### 4.2. Obtaining the Anthocyanins Extract (AEE)

Elderberries (*Sambucus nigra*) arrived at maturity stage were harvested at the end of August (Hunedoara, Romania). Two vouchers herbarium specimens no. 530, respective531 were deposited at Department of Pharmaceutical Botany (University of Medicine and Pharmacy, Cluj-Napoca, Romania). Anthocyanin and non-anthocyanin compounds were extracted by homogenization of elderberries (5 g) until becoming colorless in acidified methanol (0.3% HCl (*v*/*v*)) using the ultraturax (Miccra D-9 KT Digitronic, Bergheim, Germany), then stored at 4 °C in the dark for 24 h. The extract (EE) was concentrated under reduced pressure at 35 °C and then subjected to solid phase extraction procedure. Briefly, the aqueous extract was applied to a C-18 Sep-Pak cartridge and anthocyanins and other polyphenols were adsorbed onto the column, while other water-soluble compounds—such as sugars and acids—were removed by washing the column with acidified water (0.01% HCl *v*/*v*)). The second fraction, containing polyphenols (other than anthocyanins), was subsequently eluted with ethylacetate. Cyanidin-based anthocyanins were eluted with acidified methanol (0.01% HCl *v*/*v*)) and methanol was evaporated by using a rotatory evaporator at 35 °C.

### 4.3. LC/DAD/ESI-MS Analysis of AEE

An Agilent Technologies 1200 LC system (Chelmsford, MA, USA) equipped with G1311A Quaternary Pump, G1322A degasser, G1329A autosampler and G1315D photo-diode array (DAD) detector was used for the identification of cyanidin-based anthocyanins isolated from AEE. Separation was achieved on a Luna Phenomenex C-18 column (5 μm, 25 cm × 4.6 mm). The temperature of the column oven was set at 25 °C. Elution was performed using the flowing gradient of mobile phase A (4.5% formic acid in double distilled water) and mobile phase B (acetonitrile), with a solvent flow rate set at 0.5 mL/min. The gradient elution system started with 10% B for 9 min. The percent of B increased to 12% at 17 min and continued up to 25% B at min 20. A full spectrum was recorded. Using the same conditions mentioned above, an LC Quadrupole 6110 mass spectrometer system (Agilent Technologies, Chelmsford, MA, USA) equipped with an ESI probe was used to confirm the identification of cyanidin-based anthocyanins. The ESI parameters were the following: positive mode, tuning with Cy-3-*O*-samb; nebulizer pressure, 40 psi; N_2_ drying gas, 10 mL·min^−1^; drying gas temperature, 350 °C. Ions scan ranged from 260 to 1000 *m/z*. Peak assignments were made based on retention time, UV-visible spectra and fragmentation ion obtained by LC-DAD and LC-ESI-MS analyses. Quantification of total anthocyanins from AEE was done using a calibration curve performed with Cy-3-*O*-samb standard and was expressed as mg Cy-3-*O*-samb/ 100 g fresh fruit. HPLC chromatograms were recorded at 520 nm for anthocyanin identification. For cell culture studies, a higher amount of fruit samples have been extracted, fractionated, and concentrated. The more fractionations were done in order to have a more concentrated AEE stock solution obtained, which was expressed as it resulted from calibration curve concentration of 5700 μg Cy-3-*O*-samb/mL extract, with 71% purity being used for preparing the concentrations delivered to cell culture.

### 4.4. Cell Culture

Metastatic B16-F10 murine melanoma cell line was obtained from American Type Culture Collection (Rockville, MD, USA). B16-F10 cells were grown in standard conditions with cell culture medium DMEM containing 1g/L glucose, 10% FBS, 2 mM glutamine, 1% penicillin and streptomicin. B16-F10 cells were subcultured every three days and used for experiments at passages 18–30.

### 4.5. Analysis of Cell Viability

B16-F10 cells (8 × 10^3^ cells/well) were seeded on 96-well plate and cultured in DMEM containing 10% FBS for 24 h. The medium was then replaced with complete medium containing or not AEE at various concentrations (0–500 μg/mL) for 24 h at 37 °C and 5% CO_2_. AEE stock solution (5700 μg/mL) was prepared with serum-free medium containing 0.3% DMSO. Prior to each experiment, AEE was diluted to final concentrations in the complete culture medium. AEE treatment was applied for 24 h at 37 °C and 5% CO_2_. To assess the cell viability after AEE treatment, a previously reported procedure was followed [19]. Briefly, cell culture medium was removed and the MTT reagent in HBSS (Hank’s buffered salt solution) buffer (0.5 mg/mL) was added to each well. After 2 h of incubation, the MTT solution was removed and the formazan crystals were dissolved in DMSO. The solubilized formazan formed in viable cells was measured at 550 and 630 nm (for sample and background, respectively) using the microplate reader HT BioTek Synergy (BioTek Instruments, Winooski, VT, USA). The results were expressed as survival percent with respect to an untreated control.

### 4.6. Detection of LDH Activity

The cytotoxicity of AEE was evaluated in a 96-well plate, by measuring the total LDH activity in B16-F10 cell lysates. AEE treatment was applied on B16-F10 cells in free serum media for 24 h. Lysates B16-F10 cells (8 × 10^3^ cells/well) were centrifuged at 250 g for 4 min at room temperature. Supernatants (50 μL) were transferred into new wells and the enzymatic analysis was done according to the manufacturer’s instructions of the In Vitro Toxicology Assay Kit, LDH based (TOX7 Sigma, St. Louis, MO, USA). Absorbance values measured at 490 nm, using the BioTek Synergy HT microplate reader (BioTek Instruments, Winooski, VT, USA) were translated into LDH leakage percent of untreated cells.

### 4.7. 96-Well-Based EB/AO Staining

B16-F10 cells (8 × 10^3^ cells/well) were seeded on 96-well plate and cultured in DMEM containing 10% FBS for 24 h. The medium was then replaced with complete medium containing AEE (250 μg/mL). After an incubation of 24 h, B16-F10 cells were stained according to a previously reported method for apoptotic quantification [29]. Briefly, the B16-F10 cells were centrifuged at 129 g for 5 min using the Eppendorf 5804R centrifuge with inserts for 96-well plates. EB/AO dye mix (8 μL) was added to each well, and cells were viewed under the Carl Zeiss Observer A1 microscope, with AxioVision image processing software, Jena, Germany).

### 4.8. TUNEL Assay

B16-F10 cells (8 × 10^4^ cells/well), cultured on two-well chamber slides, were treated with AEE (250 μg/mL), for 24 h. An untreated sample was also used as control. Prior to confocal microscopy TUNEL assay B16-F10 cells were fixed with 4% paraformaldehyde for 15 min. The slides were processed for TUNEL assay using ApopTag Red In Situ Apoptosis Detection Kit (Chemicon, Millipore, Bedford, MA, USA) according to the manufacturer’s instructions. The samples were counterstained with 5 μM Draq5 in distilled water for 5 min at room temperature, followed by washing with PBS. Fluorescent images were acquired with a Zeiss LSM 710 confocal laser scanning unit (Oberkochen, Germany) equipped with argon and an HeNe laser mounted on an Axio Observer Z1 Inverted Microscope. The number of TUNEL-positive cells per 1000 cells was counted in various areas and expressed as a percentage of total cells counted. The percentage of apoptotic cells from the total tumor cells was expressed as apoptotic index.

### 4.9. Statistical Analysis

All data were expressed as mean ± standard error of mean (SEM) for each sample analyzed three times. Analysis of variance (ANOVA) and Duncan multiple range tests were used to determine significant differences between values (*p* < 0.05).

## 5. Conclusions

Apoptosis of tumor cells is a recently preferred method to attenuate or control the tumor progression. Anthocyanins are known to have such an effect on tumor cells. However, there are several drawbacks that need to be overcome before using them as potentially therapeutic agents. Therefore, one of the practical strategies to increase the bioavailability of anthocyanins could be their topical administration, thus directly attenuating the ability of cancer cells to resist apoptosis. Nevertheless, our data indicate that anthocyanins could reduce melanoma proliferation and induce apoptosis, so they could be potentially used as treatment adjuvants for a topical administration. Considering that the extraction method used in this study is suitable to obtain an enriched cyanidin-based anthocyanins fraction, we cannot exclude the contributions of impurities like phenolic acids or flavonols. Further, we consider that the in vivo investigation is of high relevance in order to determine the effect of the topical application of anthocyanins on tumor cells.

## Figures and Tables

**Table 1:** Identification of cyanidin-based anthocyanins, their retention times, molecular ions by mass spectrometry (MS) fragmentation, the UV-VISabsorption maxima and the individual anthocyanins content of anthocyanins enriched extract (AEE), calculated as Cy-3-*O*-samb equivalent (mg/100 g of fresh weight (FW)).

Peak	HPLC *R*_t_ (min)	λ_max_ (nm)	Molecular Ion (MS) *m*/*z*^+^	Fragment (MS-MS) *m*/*z*^+^	Identification of Anthocyanins	Anthocyanins Content (mg/100g FW)
1	4.7	275 (525)	743	581,449,287	Cy-3-*O*-samb-5-gluc	48.49 ± 0.74
2	6.8	275 (516)	611	449,287	Cy-3,5-digluc	84.57 ± 1.53
3	10.4	271 (518)	581	449,287	Cy-hexoside-pentoside	35.34 ± 1.42
4	12.2	279 (518)	581	449,287	Cy-3-*O*-samb	255.56 ± 2.23
5	13.0	275 (517)	449	287	Cy-3-*O*-gluc	71.18 ± 1.83
					Total	495.16 ± 7.75

## Abbreviations

IL-6	Interleukin 6
TNF-alfa	Tumor Necrosis Factor alfa
MAP kinases	Mitogen-activated protein kinases
Erk 1/2	Extracellular signal-regulated kinase 1/2
p38	Stress-activated protein kinase p38
JNK 1/2	c-Jun *N*-terminal kinase
MKK4	Mitogen-activated protein kinase kinase
NF-κB	Nuclear factor NF-κB protein
AP-1	Activator protein-1
FBS	Fetal bovine serum
DMEM	Dulbecco’s Modified Eagle Medium
MTT	3-(4,5-Dimethylthiazol-2-yl)-2,5-Diphenyltetrazolium bromide
MeOH	Methanol
EtOAc	Ethylacetate
HCl	Hydrochloric acid
DMSO	Dimethylsulfoxide
EB/AO	Ethidium Bromide/Acridine Orange
AEE	Anthocyanins enriched extract
LDH	Lactate Dehydrogenase
TUNEL assay	Terminal deoxynucleotidyl transferase dUTP nick end labeling assay
Draq5	1,5-Bis{[2-(di-methylamino) ethyl]amino}-4,8-Dihydroxyanthracene-9,10-Dione
PBS	Phosphate Buffered Saline
LC/DAD/ESI-MS	Liquid Chromatography/Diode Array/Electrospray Ionization-Mass Spectrometry
Cy	Cyanidine
Cy-3-*O*-samb-5-gluc	Cyanidine-3-*O*-sambubioside-5-glucoside
Cy-3,5-*O*-gluc	Cyanidine-3,5-glucoside
